# Is there a place for Tooth Mousse® in the prevention and treatment of early dental caries? A systematic review

**DOI:** 10.1186/s12903-015-0095-6

**Published:** 2015-09-25

**Authors:** Sarah Raphael, Anthony Blinkhorn

**Affiliations:** Department of Population Oral Health, Faculty of Dentistry, The University of Sydney, 1 Mons Road, Westmead, NSW 2145 Australia; Colgate Palmolive Pty. Ltd, 345 George Street, Sydney, NSW 2000 Australia

**Keywords:** Tooth Mousse, Tooth Mousse Plus, Dental Caries, MI Paste, MI Paste Plus, Casein phosphopeptide-amorphous calcium phosphate (CPP-ACP), Clinical Trials

## Abstract

**Background:**

It is important for Dental Professionals to consider the evidence for the effectiveness of the preventive strategies used to maintain good oral health and reduce the risk of caries in their patients. Whilst many of the traditional preventive activities, including the recommendation and use of fluoride products and the placement of fissure sealants have a wealth of clinical evidence to support their use, some of the newer preventive agents have a more limited evidence base. In order to investigate the level of scientific support behind one such technology, a systematic literature review was carried out to assess the effectiveness of Tooth Mousse® (MI Paste®) and Tooth Mousse Plus® (MI Paste Plus®) in the prevention and treatment of early dental caries.

**Methods:**

A broad search strategy using Medline via OvidSP and EMBASE was performed in order to capture all published studies to related Casein Phosphopeptide-Amorphous Calcium Phosphate. In addition to the above searches the terms “CPP ACP” and “casein phosphopeptide amorphous calcium phosphate” were searched using PREMEDLINE and the Cochrane Central Register of Controlled Trials. Inclusion criteria were clinical trials of participants of any age, comparing the use of Tooth Mousse® (MI Paste®) or Tooth Mousse Plus® (MI Paste Plus®) to a routine oral care regimen and reporting recognised clinical outcome measures for early caries lesions. Only research studies in English were selected.

**Results:**

7576 articles were identified, but the majority were duplicates. Once these were removed 172 articles were inspected and the focus on ‘CPP-ACP formulations of Tooth Mousse® (MI Paste®) and Tooth Mousse Plus® (MI Paste Plus®) resulted in 29 articles being selected, and of these 12 studies met the inclusion criteria and were considered acceptable for the systematic review.

**Discussion:**

The overall findings of this review did not show any significant benefits of using Tooth Mousse® (MI Paste®) products over brushing with a fluoride toothpaste for the prevention of early dental caries. With regard to the regression of white spot lesions in orthodontic patients there is a tendency towards a benefit for the use of Tooth Mousse® (MI Paste®) but the quality of evidence is limited. There is a lack of evidence to support the use of Tooth Mousse Plus® (MI Paste Plus®) over Tooth Mousse® (MI Paste®) at this time.

**Conclusion:**

This review suggests that further well-designed randomized controlled trials are required prior to the widespread recommendation of Tooth Mousse® products for the prevention and treatment of early dental caries in the general population.

**Electronic supplementary material:**

The online version of this article (doi:10.1186/s12903-015-0095-6) contains supplementary material, which is available to authorized users.

## Background

Forty years ago dental caries was a major health problem for most children and adults living in developed countries and the dental profession was unable to cope with the demand for clinical care [1]. Since then the prevalence and severity of dental caries has declined. For example the mean DMFT for 12 year olds in Australia dropped from 4.8 in 1977 to 1.1 in 1993 [2] and in the United Kingdom from 3.1 in 1973 to 0.8 in 2003 [3]. The change in caries prevalence has been accompanied by an alteration in the distribution of lesions, with pit and fissure caries levels increasing [4]. Despite the general improvements in oral health, caries continues to be a challenge for the dental team, particularly for those clinicians working in low income and socially disadvantaged areas where the prevalence of caries is still a public health issue. Another change that has had an impact on clinical practice is the increased prevalence of new carious lesions in adults, reaching a level as high as that seen in children [5]. Therefore, the profession has to plan treatment and preventive care pathways based on the understanding that dental caries is no longer a rapidly developing problem in childhood, but a slowly progressing disease of adulthood.

The general decline in dental caries that has occurred may have led to some complacency amongst the dental team when considering the impact preventive care can have on patients. This conundrum is demonstrated in a study which found that 25 % of children initially caries free developed caries over the following three years and those with one carious lesion were five times more likely to develop more lesions when compared with those free of the disease [6]. Therefore professionals who only provide preventive advice to those with dental caries will be doing a disservice to many patients.

Given that oral health care advice is a key part of the dental service for patients it is important to consider the evidence for the effectiveness of our preventive activities. We want to be confident we can maintain good oral health and reduce the risk of caries.

There are four potential preventive strategies which can be used by the dental team, namely

- Regular disturbance of the plaque biofilm by brushing twice a day with a fluoride toothpaste [7]. Other fluoride agents may also be added if the caries risk warrants their use [8–11].
- Reduction in the frequency of consumption of refined carbohydrate [12].
- Placement of pit and fissure sealants, to address the increase in occlusal caries [13].
- Regular monitoring of early carious lesions to check for progression and determine if fluoride products are being used appropriately. Reinforcement of lifestyle changes such as controlling the frequency of sugar consumption and brushing with a fluoride toothpaste twice a day [14].

It is clear that there is a wealth of scientific evidence supporting these preventive strategies, especially the use of fluorides. However research scientists have also investigated other agents which could be of value in helping the dental team and their patients to control dental caries. Ones which have achieved great popularity are Tooth Mousse® (MI Paste®) and Tooth Mousse Plus® (MI Paste Plus®) containing the active ingredient casein phosphopeptide – amorphous calcium phosphate (CPP-ACP) and are marketed by the GC Corporation.

These products are based on the pioneering work of Professor Eric Reynolds and his team at the University of Melbourne Dental School [15], who developed Recaldent®(CPP-ACP technology). Tooth Mousse® (MI Paste®) contains 10 % of the Recaldent® molecule by weight. Calcium phosphopeptide (CPP) is a milk derived protein able to bind calcium and phosphate ions, and stabilise them as amorphous calcium phosphate (ACP). CPP-ACP adheres intra-orally to plaque pellicle, hydroxyapatite as well as soft tissues. It supplies bioavailable calcium and phosphate into saliva and plaque fluid enabling it to drive remineralisation [16]. *In vitro* studies demonstrate that when placed on the surface of a tooth, CPP-ACP interacts with hydrogen ions and can diffuse into enamel where it produces subsurface mineral gains [17].

Tooth Mousse Plus® (MI Paste Plus®) contains 900 parts per million fluoride in a molar ratio with the calcium and phosphate of 5 calcium, 3 phosphate and 1 fluoride which is reported by Reynolds and co-workers as the ideal ratio for building fluorapatite [18, 19].

The development of the GC products Tooth Mousse® (MI Paste®) and Tooth Mousse Plus® (MI Paste Plus®) is to be applauded as scientific innovation is critical in the quest to improve the oral health of patients. However when the dental team use and recommend products for patient care there must be sound scientific evidence to support their treatment planning decisions and advice. CPP-ACP in the form of Tooth Mousse® (MI Paste®) and Tooth Mousse Plus® (MI Paste Plus®) are widely recommended for the prevention of early dental caries. The manufacturer instructions recommend Tooth Mousse® (MI Paste®) for patients of any age except those with milk protein allergies but limits the indication of Tooth Mousse Plus® (MI Paste Plus®) to patients over six years of age because of the fluoride content. These products are much more expensive to use than fluoride products, so it is important to examine the evidence supporting their general usage. To this end a systematic review on the specific use of these two products for caries prevention and treatment has been undertaken, in order to determine whether their efficacy warrants use in general dental practice.

The aim of the systematic review is to answer the question. “Is there sufficient clinical evidence available to support the use of Tooth Mousse® (MI Paste®) and Tooth Mousse Plus® (MI Paste Plus®) over a routine oral care regimen for the prevention and treatment of early dental caries?”

## Methods

A broad search strategy using Medline via OvidSP and EMBASE was performed in order to capture all published studies to related Casein Phosphopeptide-Amorphous Calcium Phosphate (See Additional file 1). In addition to the above searches the terms “CPP ACP” and “casein phosphopeptide amorphous calcium phosphate” were searched using PREMEDLINE and the Cochrane Central Register of Controlled Trials. From these searches – one record from PREMEDLINE was identified as relevant to this review, whilst the Cochrane Database of Systematic Reviews identified a protocol for a systematic review entitled “Non fluoride topical remineralising agents containing calcium and/or phosphate for controlling dental caries [20]. This review by the Cochrane collaboration aims to evaluate non-fluoride topical remineralising agents containing any formulation of calcium and/or phosphate at any concentration and in any topically-applied delivery vehicle and as such has a broader scope than the focus of this review. The inclusion and exclusion criteria used to filter the identified studies can be found in Table 1.

**Table 1:** Inclusion and Exclusion criteria

Inclusion criteria	Exclusion criteria
Participants: People of any age or gender at risk of dental caries.	Reviews, case reports, abstracts, letters to editors, editorials, commentaries, *in vitro* and *in situ* studies utilising bovine or human enamel were excluded.
Interventions: The use of Tooth Mousse® (MI Paste®) or Tooth Mousse Plus® (MI Paste Plus®) in accordance with the manufacturer’s instructions for the prevention or treatment of dental caries.	Non-english language studies were excluded.
Comparisons: Tooth Mousse® (MI Paste®) or Tooth Mousse Plus® (MI Paste Plus®) (Test) versus a routine oral care regimen for the prevention of dental caries (Control) with or without comparisons to additional preventive products.	Studies utilising an artificial caries model or enamel demineralization model were excluded.
Outcomes: Recognised clinical measures of early caries lesions or enamel demineralisation including - enamel microhardness, DIAGNOdent readings, QLF measurements, clinical caries scoring and visual inspection of photographic images.	Interventions: Only Tooth Mousse® (MI Paste®) or Tooth Mousse Plus® (MI Paste Plus®) formulations were considered. No other formulations of CPP-ACP such as gum, lozenges, solutions, mouthrinses, toothpastes or varnishes were considered.
Study Design: Clinical trials	Studies in which Tooth Mousse® (MI Paste®) or Tooth Mousse Plus® (MI Paste Plus®) was not used in accordance with the manufacturer’s directions for use were excluded.

The Cochrane Collaboration’s tool for assessing risk of bias was utilized in the analysis [21]. The papers included in the final review were assessed independently by both authors (SR and AB) for risk of bias.

## Results

A broad search of the literature was carried out in December 2013 that identified 7576 articles of which the majority were duplicates. Once these duplicates were removed and the remainder limited to those where CPP-ACP was the primary focus and published in English, 172 articles were identified for closer inspection. The PREMEDLINE search identified one additional paper. Of the 173 articles, the inclusion and exclusion criteria in Table 1 were applied which led to 28 articles being excluded immediately as they were review articles, case reports or letters to the editor.

**Fig. 1:**
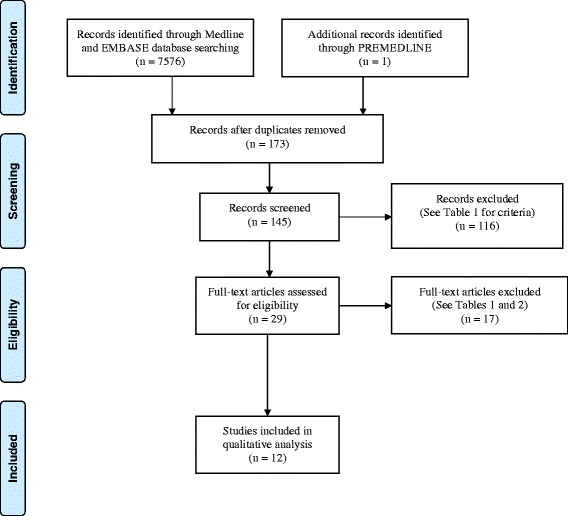
Flowchart of systematic review process

All of the 145 articles remaining were studied by title and abstract as an initial filter. As the current systematic review is focussed entirely on the CPP-ACP formulations of Tooth Mousse® (MI Paste®) and Tooth Mousse Plus® (MI Paste Plus®) – all studies employing any other formulations of CPP-ACP including solutions, chewing gum and dentifrice were excluded. This filter decreased the number of articles to 29. These 29 articles were studied in full text, nine of which were excluded as they were either *in vitro* studies or *in situ* studies utilising bovine enamel, giving 20 studies dating from 2007 to 2013 for final review (Fig. 1). Eight of the twenty studies were excluded – the titles and reasons for exclusion are summarised in Table 2. Andersson *et al.* [22] did not use Tooth Mousse® (MI Paste®) or Tooth Mousse Plus® (MI Paste Plus®) but instead utilised a proprietary CPP–ACP dental crème which is either no longer readily available or has been discontinued. Robertson *et al*. [23] delivered the MI Paste Plus® in preformed trays, which does not follow the manufacturer’s directions for use. The remaining six [24–29] studies were found on closer examination to have used outcome measures not recognised as clinical care measures or employed artificially demineralised tooth substance in an *‘in situ’* model which were exclusion criteria (See Table 1). Both authors (SR and AB) reviewed the final 20 studies independently and reached consensus on which papers were included in the final review. Of the 12 studies available for this systematic review, three studies reported on prevention [30–32] (Table 3) and nine reported on the treatment or regression of caries [33–41] (Table 4).

**Table 2:** Studies excluded from the final review, with reasons for exclusion

Author	Title	Reason for exclusion
Andersson *et al.* [22] 2007	Effect of a dental cream containing amorphous calcium phosphate complexes on white spot lesion regression assessed by laser fluorescence.	Product used - a CPP-ACP dental crème which is difficult to obtain or no longer available commercially.
Robertson *et al. *[23] 2011	MI Paste Plus® to prevent demineralisation in orthodontic patients. A prospective randomised controlled trial.	Product delivery – did not follow the recommended usage instructions for the product.
		Participants wore customised intra-oral trays containing MI Paste Plus® for a minimum of 3–5 mins per day.
Kitasako *et al. *[24] 2010	The clinical application of surface pH measurements to longitudinally assess white spot enamel lesions.	Outcome measure - surface pH of enamel.
Marchisio *et al. *[25] 2010	Salivary pH level and bacterial plaque evaluation in orthodontic patients treated with Recaldent® products.	Outcome measure - salivary and plaque pH.
Thepyou *et al.* [26] 2013	Casein phophopeptide-amorphous calcium phosphate and glass ionomer show distinct effects in the remineralization of proximal artificial caries lesion *in situ*.	Test specimens - artificial caries lesions.
Caruana *et al.* [27] 2009	The effect of casein and calcium containing paste on plaque pH following a subsequent carbohydrate challenge.	Outcome measure - plaque pH.
Ferrazzano *et al.* [28] 2011	*In vivo* remineralising effect of GC Tooth Mousse® on early dental enamel lesion: SEM analysis.	Test specimens – artificially demineralised enamel.
Baroni *et al.* [29] 2014	A SEM and non-contact surface white light profilometry *in vivo* study of the effect of a crème containing CPP-ACP and fluoride on young etched enamel.	Outcome measure *-* incisor surface morphology using scanning electron microscope and white light profilometry following etching with 37 % phosphoric acid.

**Table 3:** Prevention Studies included in the Final Review

Author & Publication Year	Title	Fluoride Exposure	Study design	Study population	Assessment	Results
Uysal *et al*. [30] 2010	Effects of different topical agents on enamel demineralization around orthodontic brackets: *and in vivo* an in *vitro study* ^a^	Unspecified water fluoridation. All groups used non-fluoridated toothpaste.	Single-blind randomized clinical study on the prevention of white spot lesions.	21 orthodontic patients aged 13–17 years	Enamel microhardness	*In vivo* results showed no statistical difference between the two topical agents. Both topical agents showed statistically significant difference from the control group after 60 days.
			Topical agents – Tooth Mousse®, Fluoridin N5 5 % (22,600 ppm) sodium fluoride topical gel and control group. Application of Tooth Mousse® or Fluoridin N% gel carried out on teeth with orthodontic brackets scheduled for extraction after 60 days. No application on patients in the control group.			
Sitthisettapong *et al.* [31] 2012	Effect of CPP-ACP paste on dental caries in primary teeth: A randomized trial	Non-fluoridated water. Fluoride toothpaste (1000 ppm) used by both groups.	Double-blind randomized placebo-controlled clinical trial on the prevention of caries in high caries risk pre-school children. Topical agents – Tooth Mousse® or placebo paste.	296 pre-school children aged 2½ to 3½ years	Caries lesion transitions (regression, stability or progression) using ICDASII and dmfs	No significant difference was observed between Tooth Mousse® group and placebo group after 1 year
			Paste applied every school day by trained teacher, following toothbrushing.			
Plonka *et al.* [32] 2013	A randomized controlled clinical trial comparing a remineralizing paste with an anti-bacterial gel to prevent early childhood caries	Unspecified water fluoridation. Fluoride toothpaste (400 ppm) used by all groups.	Randomized controlled trial comparing Tooth Mousse®, chlorhexidine gel and 0.304 % (400 ppm) fluoride toothpaste for reducing mutans streptococci colonization and preventing early childhood caries.	622 infants aged 0 to 24 months	Cavitations and white spot lesions of early caries	No significant difference in incidence between the three groups after 24 months.
			Tooth Mousse® and chlorhexidine gel were applied by child’s mother in the evening following toothbrushing.

**Table 4:** Regression Studies included in the Final Review

Author & Publication Year	Title	Fluoride Exposure	Study design	Study population	Assessment	Results
Bailey *et al.* [33] 2009	Regression of post-orthodontic lesions by a remineralizing cream	Optimal water fluoridation.	A double-blind randomized clinical trial to test whether more white spot lesions would regress in participants using Tooth Mousse® than a placebo paste. Patients applied Tooth Mousse® or placebo paste twice daily following toothbrushing for 12 weeks.	45 adolescents aged 12 to 19 years immediately following debonding of fixed orthodontic appliances.	ICDAS II clinical scoring	No statistically significant difference in the transition scores between the intervention and control groups was found overall. Regression of lesions with severity codes 2 or 3 at baseline in the Tooth Mousse® group were statistically significant higher than the placebo group at 12 weeks.
		Both groups used fluoride toothpaste (1000 ppm) and supervised fluoride mouthrinses (900 ppm) given at each assessment visit to both groups.				
Altenburger *et al.* [34] 2010	The evaluation of fluorescence changes after application of casein phophopeptides (CPP) and amorphous calcium phosphate on early carious lesions.	Unspecified water fluoridation.	A single-blind randomized clinical study to test the daily application of Tooth Mousse® to remineralize initially demineralized enamel fissures compared to a control group. Patients in test group applied Tooth Mousse® once daily onto the occlusal surface of teeth.	32 subjects aged 22 to 31 years with molars and premolars with DIAGNOdent readings between 15 and 20.	DIAGNOdent readings and visual classification according to Ekstrand *et al.* [42]	There was a statistically significant difference between the DIAGNOdent reading in the test and control groups after 2 and 3 weeks. No statistical difference was found between the groups using the visual classification.
		Fluoride toothpaste (1450 ppm) used by both groups.				
Beerens *et al.* [35] 2010	Effects of casein phophopeptide amorphous calcium fluoride phosphate paste of white spot lesions and dental plaque after orthodontic treatment: a 3-month follow-up.^b^	Unfluoridated water.	A double-blind randomized clinical trial to investigate the effects of Tooth Mousse Plus® on dental plaque and on the remineralization of enamel white spot lesions compared to a control group.	65 adolescents 12–19 years of age immediately following the removal of fixed orthodontic appliances.	Quantitative light-induce fluorescence (QLF) images and plaque samples.^b^	Significant improvement in lesion depth was observed in both groups. No significant difference was found between the test and control groups after 12 weeks.
		Fluoride toothpaste (unspecified concentration) used by both groups.				
			Patients applied Tooth Mousse Plus® or placebo paste once daily before bedtime.			
Brochner *et al*. [36] 2011	Treatment of post-orthodontic white spot lesions with casein phosphopeptide-stabilised amorphous calcium phosphate.	Low water fluoridation (<0.2 ppm).	A randomized single-blind clinical study to investigate the effect of daily applications of Tooth Mousse® on white spot lesions compared to a control group.	60 adolescents aged 13–18 years immediately following the removal of fixed orthodontic appliances.	Visual inspection of photographs and Quantitative light-induced fluorescence (QLF) measurements.	Statistically significant regression of white spot lesions measured by visual inspection and QLF was found in both the intervention and control groups. There was no significant difference between the groups after 4 weeks.
		Fluoride toothpaste (1100 ppm) used once daily in the test group and twice daily in the control group.				
			Patients in the test group applied Tooth Mousse® once daily in the evening and brushed with fluoride toothpaste in the morning. Patients in the control group brushed twice daily with fluoride toothpaste.			
Wang *et al.* [37] 2012	Clinical evaluation of remineralization potential of casein phosphopeptide amorphous calcium phosphate nanocomplexes for enamel decalcification in orthodontics.	Unspecified water fluoridation. Test group used non-fluoridated toothpaste. Control group used fluoride toothpaste (1100 ppm).	A single-blind clinical study to evaluate the remineralizing effect of Tooth Mousse® versus twice-daily brushing with fluoride toothpaste on enamel decalcification in orthodontics. Randomization of test and control group was not reported.	40 adolescents aged below 18 years of age undergoing fixed orthodontic appliance therapy.	Visual inspection of photographs scored using an enamel decalcification index (EDI).	Statistically significant reductions in the EDI of the Tooth Mousse® group were found. No statistically significant reduction of EDI was reported in the fluoride toothpaste group during the 6 month study.
			Patients in the test group applied Tooth Mousse® once daily following evening toothbrushing with non-fluoride toothpaste. Patients in the control group brushed twice daily with fluoride toothpaste			
Akin & Basciftci [38] 2012	Can white spot lesions be treated effectively?	Unspecified water fluoridation.	A prospective clinical controlled study to determine the effectiveness of 0.025 % (100 ppm) sodium fluoride mouthrinse, Tooth Mousse® and the microabrasion technique in reducing white spot lesions compared with a control group. Randomization of test and control groups and blinding was not reported. Patients in the Tooth Mousse® group applied the crème twice daily after toothbrushing with fluoride toothpaste. Patients in the mouthrinse group rinsed for 30 s twice daily after brushing with fluoride toothpaste. In the microabrasion group the procedure was performed with a 18 % hydrochloric acid/pumice mixture and was repeated four or five times. Patients in the control group brushed their teeth (toothpaste not specified).	80 adolescents with post-orthodontic demineralized lesions.	Digital photographic images.	Statistically significant reductions in the extent of white spot lesions occurred in all groups. Microabrasion, followed by Tooth Mousse® showed the highest success rates for the postorthodontic remineralization over a 6 month study period.
		The Mouthrinse and Tooth Mousse Groups used fluoride toothpaste (unspecified concentration). The Control group brushed their teeth (toothpaste unspecified) and there was no reporting of toothbrushing in the Microabrasion group				
Krithikadatta *et al*. [39] 2013	Remineralisation of occlusal white spot lesions with a combination of 10 % CPP-ACP and 0.2 % sodium fluoride evaluated using DIAGNOdent: A pilot study.	Unspecified water fluoridation. All groups had standardised diet and oral hygiene practices (use of fluoride toothpaste was unspecified).	A randomzed single-blind clinical study to evaluate the efficacy of Tooth Mousse®, Tooth Mousse Plus® compared to 0.5 % fluoride mouthrinse for the remineralisation of occlusal white spot lesions. Patients in the Tooth Mousse® and Tooth Mousse Plus® groups applied the respective crèmes twice daily following toothbrushing. Patients in the mouthrinse group rinsed once daily for 30 s.	45 dental students aged 17–20 years.	DIAGNOdent readings and visual classification according to Ekstrand *et al.* [42]	All 3 groups showed highly significant remineralising potential at 30 days. Tooth Mousse® and Tooth Mousse Plus® showed significantly higher remineralisation compared to the fluoride mouthrinse group.
Vashisht *et al.* [40] 2013	Role of casein phosphopeptide amorphous calcium phosphate in remineralization of white spot lesions and inhibition of *Streptococcus mutans*?^c^	Unspecified water fluoridation. Both groups used fluoride toothpaste (1450 ppm).	A randomized clinical study to evaluate the remineralizing effect of Tooth Mousse® on white spot lesions compared with a control group. Blinding of the examiner was not reported. Patients in the Tooth Mousse® group applied the crème twice daily after toothbrushing. Patients in the control group brushed twice daily.	60 adolescents undergoing orthodontic treatment	DIAGNOdent readings and visual classification using ICDAS II clinical scoring	There was a statistically significant increase in DIAGNOdent readings in the control group from baseline after 3 months. There was no significant difference found in the DIAGNOdent readings in the test group from baseline to 3 months.
Huang *et al.* [41] 2013	Effectiveness of MI Paste Plus and PreviDent fluoride varnish for the treatment of white spot lesions: A randomized controlled trial.	Unspecified water fluoridation. All groups used fluoride toothpaste (1100 ppm).	A randomized single-blind parallel group trial comparing the effectiveness of daily application of MI Paste Plus® for 8 weeks with a single application of 5 % sodium fluoride varnish to a control group in improving the appearance of white spot lesions after orthodontic treatment.	115 adolescents aged between 12 and 20 years following orthodontic treatment.	Visual assessment using photographic records performed by dental experts, lay persons and the patients.	No significant differences were found in the test groups compared to the control group at the end of the 8 week study by any of the examining panels.
			Patients in the MI Paste Plus® group applied the crème twice daily. Patients in the fluoride varnish group received a single application of varnish at the start of the study. Patients in the control group followed routine oral hygiene at home.			

^a^Only the *in vivo* study was considered

^b^Only the QLF results were considered

^c^Only the remineralization results were considered

Two of the three prevention studies were double-blind randomized controlled trials in populations of pre-school children [31, 32]. These studies found no significant benefits in the use of Tooth Mousse® (MI Paste®) over standard brushing with either 1000 ppm [31] or 400 ppm [32] fluoride toothpaste. The authors concluded that there was insufficient evidence to justify the daily use of Tooth Mousse® (MI Paste®) to control dental caries in these populations. The other prevention study published by Uysal *et al*. [30] was the *in vivo* study comparing the use of Tooth Mousse® or a 5 % sodium fluoride gel with a control group to prevent white spot lesions. The results of the *in vitro* study described in this article were not considered or included in this systematic review. In the *in vivo* study, patients used a non-fluoride toothpaste and did not receive any oral hygiene instruction. This study showed a statistically significant difference in the enamel microhardness of premolar teeth extracted after the 60 day test period in both the Tooth Mousse® and fluoride gel groups, compared to the control group, but no significant difference between the test groups.

Nine studies [33–41] reported on the treatment or regression of dental caries (Table 4). In the majority of studies fluoride toothpaste was used by participants in all the study groups. However, one study [39] did not specify whether fluoride toothpaste was used, a second study did not fully specify the use of fluoride toothpaste in all groups [38] and in another [37] a non-fluoride toothpaste was used in the test group but fluoride toothpaste was used in the control group.

All except two [34, 39] of the nine studies reported on the regression of white spot lesions in orthodontic patients. This body of evidence, containing seven clinical studies [33, 35–38, 40, 41] of variable strength of evidence utilised either visual scoring or fluorescence techniques for the assessment of dental caries. Some were compared with placebo pastes and others with different preventive products and/or control groups. Of these seven studies, four showed a significant advantage from the use of Tooth Mousse® in the regression of white spot lesions in orthodontic patients over 12 weeks to 6 months [33, 37, 38, 40].

The remaining three studies reported no significant difference between the Tooth Mousse® (MI Paste®) or Tooth Mousse Plus® (MI Paste Plus®) group and control/placebo group over periods of four weeks to three months [35, 36, 41].

Altenburger *et al*. [34] reported the remineralization of demineralized enamel fissures in 32 young adults using Tooth Mousse® using both laser fluorescence and visual classification. The Tooth Mousse® group showed significant improvements in laser fluorescence but no difference was noted by the visual scoring at 2 and 3-weeks when compared to the control group.

Krithikadatta *et al*. [39] carried out a pilot study on 45 adolescent dental students with occlusal white spot lesions, comparing Tooth Mousse® and Tooth Mousse Plus® to a 0.5 % fluoride mouthrinse. All three groups showed highly significant remineralising potential over the 30 day test period, but Tooth Mousse® and Tooth Mousse Plus® were significantly more effective than the fluoride mouthrinse.

Of the twelve studies included in this systematic review, three studies were direct comparisons of Tooth Mousse® versus a control group [34, 36, 40] and two studies versus a placebo crème [31, 33]. The remaining six studies compared the efficacy of Tooth Mousse® (MI Paste®) and/or Tooth Mousse Plus® (MI Paste Plus®) to other products and techniques with or without a control group - including fluoride toothpaste [37], fluoride mouthrinse [38, 39], fluoride gel [30], fluoride varnish [41], chlorhexidine gel [32] and microabrasion [38]. The studies by Beerens *et al.* [35], Krithidkadatta *et al*. [39], and Huang *et al.* [41] involved comparisons with Tooth Mousse Plus® (MI Paste Plus®) - containing 900 ppm Fluoride, all other studies utilised only the non-fluoride containing Tooth Mousse® (MI Paste®).

With regard to safety, no serious side effects or adverse events were reported in any of the studies included in the final review. However, five studies [35, 37, 39–41] did not report side effects or adverse events in their papers. One study [33] recorded one participant with non-serious gastro-intestinal symptoms that were possibly related to the use of Tooth Mousse® and another [38] made the statement “although CPP-ACP had side effects” but did not make any reference to what the side effects were.

**Fig. 2:**
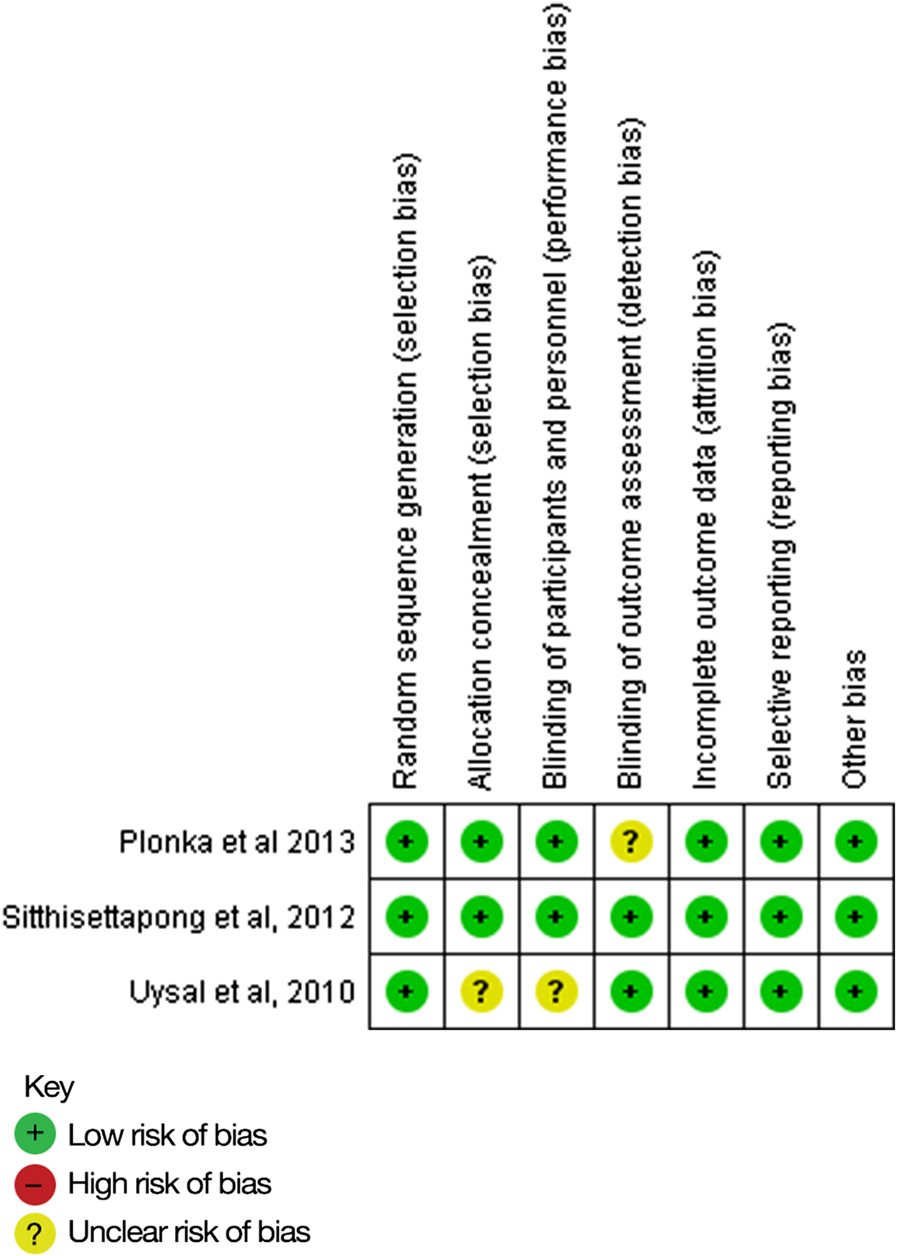
Presentation of the risk of bias assessments for the prevention studies included in the review

**Fig. 3:**
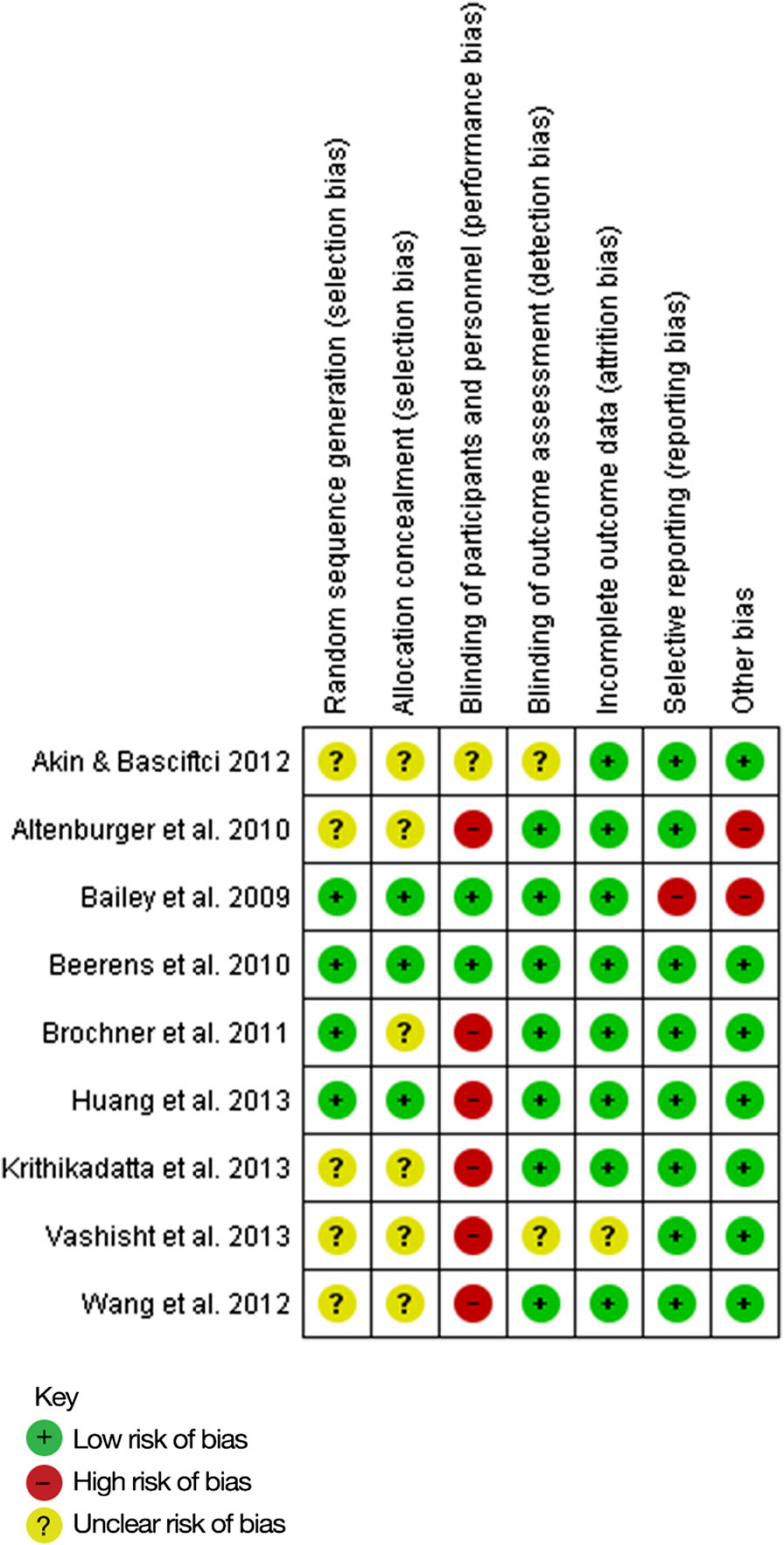
Presentation of the risk of bias assessments for the regression studies included in the review

Figure 2 indicates that the prevention studies included in our review were classified as having a low risk of bias, although questions are raised on the Uysal *et al.* [30] research on possible selection and performance bias and Plonka *et al.* [32] has a question mark over outcome assessment. The regression studies do not show such good results (Fig. 3) with over half having questionable scores for selection bias and the majority having high risk of performance bias.

The strength of evidence of the group of studies included in this systematic review are further weakened by short observation periods (five studies with duration shorter than 3 months [30, 34, 36, 38, 41]), varying outcome measures (clinical indices [31–34, 39, 40], enamel microhardness [30], laser and light-induced fluorescence [34–36, 39, 40] and visual scoring of photographs [36–38, 41]) and small number of total participants (939 participants for the three prevention studies [30–32] and 542 participants for the nine regression studies [33–41]).

## Discussion

The initial literature search on CPP-ACP yielded a huge number (7576) of publications, however a close scrutiny of the results identified 172 articles that were worthy detailed inspections. Ultimately, 20 articles were selected but eight studies (Table 2) were excluded, giving just 12 papers to consider, three focusing on prevention and nine on controlling dental caries.

If the research data for the prevention of dental caries is assessed, two of the studies [31, 32] which were randomised controlled trials (over 12 and 24 months respectively), reported that Tooth Mousse® did not offer a benefit in terms of a reduction in dental caries for young children over brushing with a fluoride toothpaste. Whilst the third prevention study [30] reported enamel demineralisation (over a relatively short period of 60 days) in a group of orthodontic patients and once again a fluoride product performed just as well as Tooth Mousse®. Therefore, it would be unwise to recommend Tooth Mousse® (MI Paste®) for the prevention dental caries.

One might also consider it somewhat unusual that the bulk of the evidence on remineralisation studies comes from orthodontic patients who are a very select group of individuals undergoing specialist dental care and not typical of the general population. However it would be unwise to dismiss the results because of the narrow specificity of the target group as it would reduce the data set to two studies. If one considers the orthodontic publications there is a some degree of evidence for the benefits of regression of white spot lesions, with four studies [33, 37, 38, 40] showing positive results and three [35, 36, 41] showing no significant difference to the control groups. When the three studies [35, 39, 41] utilising Tooth Mouth Plus® (MI Paste Plus®) are considered – Krithikadatta *et al.* [39] was the only one with a direct comparison between Tooth Mousse® and Tooth Mousse Plus®. The results of this study did not show a significant difference between the non-fluoride and fluoride-containing forms of the CPP-ACP crème and the authors suggested that further studies would be required to confirm these results.

There is a wide variation in the study designs, blinding, protocols and outcome measures in this group of studies making meta-analysis impossible. Clearly, more randomised longer-term trials are required utilizing Tooth Mousse® (MI Paste®) and Tooth Mousse Plus® (MI Paste Plus®) in accordance with the manufacturer’s instructions to clarify the benefits of use in orthodontic patients. In the general population, those individuals at high risk of developing dental caries are commonly of low socio-economic status and have less disposable income for oral care products. Whilst Tooth Mousse® (MI Paste®) has the advantage of being fluoride-free, making it suitable for use in very young children, the risk of development of fluorosis of the permanent teeth from the excessive ingestion of fluoride toothpaste is not a concern for children 6 years of age and older. The two papers [31, 32] in this review that studied the efficacy of Tooth Mousse® (MI Paste®) in children under 6 years of age do not support its use over the twice-daily use of either 1000 ppm [31] or 400 ppm [32] fluoride toothpaste. As it is also much more expensive than fluoride toothpaste the recommendation of this product in very young children cannot be supported.

With regard to the benefits of Tooth Mousse® (MI Paste®) or Tooth Mousse Plus® (MI Paste Plus®) in people 6 years of age and above, we certainly require more work to support its general use for the prevention and treatment of early caries apart from perhaps those patients undergoing orthodontic care which is often the province of more affluent individuals.

The risk of bias assessment raises important issues with the regression studies, and clearly more robust and well-executed randomised studies are required.

## Conclusions

The findings of this systematic review suggest there is a lack of evidence to support the use of Tooth Mousse® (MI Paste®) over a routine preventive fluoride regimen for the prevention of early dental caries. With regard to the use of Tooth Mousse® (MI Paste®) and Tooth Mousse Plus® (MI Paste Plus®) for the regression of white spot lesions associated with orthodontic treatment there is a tendency towards a benefit for their use but the quality of evidence is limited. Furthermore, at this time there is a lack of support for the use of fluoride-containing formulation - Tooth Mousse Plus® (MI Paste Plus®) over Tooth Mousse® (MI Paste®). New products require testing over time and the lack of sufficient high level clinical evidence for the efficacy of these specific casein phosphopeptide amorphous calcium phosphate-containing products remains a limitation. Further well-designed randomized controlled trials are required prior to the widespread recommendation of Tooth Mousse® (MI Paste®) or Tooth Mousse Plus® (MI Paste Plus®) for the prevention and treatment of early dental caries in the general population.

## Additional file

Additional file 1:
**Full search terms with numbers of articles identified.** (DOCX 15 kb)

## Abbreviations

CPP-ACP: Casein phosphopeptide-amorphous calcium phosphate
DMFT: Decayed/missing/filled teeth index
CPP: Casein phosphopeptide
ACP: Amorphous calcium phosphate
ppm: parts per million
