# Supplementary material for: Is there a place for Tooth Mousse® in the prevention and treatment of early dental caries? A systematic review
Source: BMC Oral Health. 2015 Sep 25;15:113. doi: 10.1186/s12903-015-0095-6 (PMC4583988; doi:10.1186/s12903-015-0095-6)
Supplement: Additional file 1: — Full search terms with numbers of articles identified. (DOCX 15 kb) [file 12903_2015_95_MOESM1_ESM.docx]

**Additional File – Full search terms with numbers of articles identified**

| Search Terms | Medline | EMBASE |
| --- | --- | --- |
| Casein Phosphopeptide-Amorphous Calcium Phosphate or CPP ACP | 206 | 137 |
| CPP ACP | 120 | 132 |
| Casein Phosphopeptide Amorphous Calcium Phosphate | 199 | 197 |
|  |  |  |
| CPP ACP AND dental AND decay | 2 | 3 |
| Casein Phosphopeptide Amorphous Calcium Phosphate AND dental AND decay | 4 | 4 |
|  |  |  |
| CPP ACP AND dental AND caries | 57 | 65 |
| Casein Phosphopeptide-Amorphous Calcium Phosphate AND dental AND caries | 103 | 98 |
|  |  |  |
| CPP ACP AND fluoride | 52 | 59 |
| Casein Phosphopeptide-Amorphous Calcium Phosphate AND fluoride | 100 | 97 |
|  |  |  |
| CPP ACP AND teeth | 47 | 109 |
| Casein Phosphopeptide-Amorphous Calcium Phosphate | 58 | 140 |
|  |  |  |
| CPP ACP AND mouth | 13 | 101 |
| Casein Phosphopeptide-Amorphous Calcium Phosphate AND Mouth | 22 | 156 |
|  |  |  |
| CPP ACP AND oral cavity | 1 | 1 |
| Casein Phosphopeptide-Amorphous Calcium Phosphate AND oral cavity | 3 | 2 |
|  |  |  |
| CPP ACP AND primary | 6 | 5 |
| Casein Phosphopeptide-Amorphous Calcium Phosphate AND primary | 11 | 10 |
|  |  |  |
| CPP ACP AND Permanent | 8 | 3 |
| Casein Phosphopeptide-Amorphous Calcium Phosphate AND permanent | 17 | 7 |
|  |  |  |
| CPP ACP AND paste | 40 | 41 |
| Casein Phosphopeptide-Amorphous Calcium Phosphate AND paste | 52 | 47 |
|  |  |  |
| CPP ACP AND remineralization | 84 | 72 |
| Casein Phosphopeptide-Amorphous Calcium Phosphate AND remineralization | 131 | 89 |
| CPP ACP AND remineralisation | 11 | 13 |
| Casein Phosphopeptide-Amorphous Calcium Phosphate AND remineralisation | 13 | 11 |
|  |  |  |
| CPP ACP AND demineralization | 0 | 45 |
| Casein Phosphopeptide-Amorphous Calcium Phosphate AND demineralization | 0 | 55 |
| CPP ACP AND demineralisation | 0 | 1 |
| Casein Phosphopeptide-Amorphous Calcium Phosphate AND demineralisation | 0 | 3 |
|  |  |  |
| Tooth Mousse | 42 | 81 |
| Tooth Mousse Plus | 2 | 8 |
| Tooth Mousse AND remineralization | 22 | 17 |
| Tooth Mousse AND remineralisation | 1 | 3 |
| Tooth Mousse AND demineralization | 0 | 0 |
| Tooth Mousse AND demineralisation | 0 | 0 |
|  |  |  |
| MI Paste | 27 | 95 |
| MI Paste Plus | 13 | 16 |
| MI Paste AND remineralization | 13 | 8 |
| MI Paste AND remineralisation | 0 | 0 |
| MI Paste AND demineralization | 0 | 7 |
| MI Paste AND demineralisation | 0 | 0 |
